# tugHall: a simulator of cancer-cell evolution based on the hallmarks of cancer and tumor-related genes

**DOI:** 10.1093/bioinformatics/btaa182

**Published:** 2020-03-14

**Authors:** Iurii S Nagornov, Mamoru Kato

**Affiliations:** Department of Bioinformatics, Research Institute, National Cancer Center Japan, Tokyo 104-0045, Japan

## Abstract

**Summary:**

The flood of recent cancer genomic data requires a coherent model that can sort out the findings to systematically explain clonal evolution and the resultant intra-tumor heterogeneity (ITH). Here, we present a new mathematical model designed to computationally simulate the evolution of cancer cells. The model connects the well-known hallmarks of cancer with the specific mutational states of tumor-related genes. The cell behavior phenotypes are stochastically determined, and the hallmarks probabilistically interfere with the phenotypic probabilities. In turn, the hallmark variables depend on the mutational states of tumor-related genes. Thus, our software can deepen our understanding of cancer-cell evolution and generation of ITH.

**Availability and implementation:**

The open-source code is available in the repository https://github.com/nagornovys/Cancer_cell_evolution.

**Contact:**

mamkato@ncc.go.jp

**Supplementary information:**

Supplementary data are available at *Bioinformatics* online.

## 1 Introduction

Mathematical models, often coupled with computer simulation, can provide insights into clonal evolution and intra-tumor heterogeneity in cancer, which cannot be obtained through direct observation of the genomic states of tumor patients. Previously, a branching process model that computationally simulates clonal evolution was used to estimate parameters such as the mutation rate and relative fitness of tumor subclones (Williams *et al.*, 2018). In the study, an approximate Bayesian computation (ABC) framework was applied to the observation data of variant allele frequencies (VAFs) detected by next-generation sequencing at the genome level in multiple cancers such as breast, blood, and lung cancers. Multiple studies have also reported using computer simulations for addressing cancer development and progression (Altrock *et al.*, 2015; Beerenwinkel *et al.*, 2015; Conterno Minussi *et al.*, 2019; Sottoriva *et al.*, 2013; Waclaw *et al.*, 2015; Wang *et al.*, 2014).

Despite these advances, it remains difficult to elucidate the roles of tumor-related genes in cancer development in these representative models. This is because these models postulate ‘abstract’ genes, in which generated mutations are assumed to change parameter values for the birth (cell division) and death (apoptosis) rates of tumor cells, the fitness change, and the dispersal (migration) rate. Gene functions such as angiogenesis by *VEGF* and immortalization by *TERT* are abstracted into these parameters, and so cannot directly be modeled. The roles of cancer-related genes were originally summarized under six essential ‘hallmarks’, and more recently two extra hallmarks and two characteristics were added (we will collectively refer to all the ten summary terms as hallmarks) (Hanahan and Weinberg, 2000, 2011). Such hallmarks are not only conceptually proposed, but have also been individually assigned to specific tumor-related genes in cancer knowledge databases such as COSMIC (Forbes *et al.*, 2017) and MSigDB (Liberzon *et al.*, 2015).

Introducing these essential cancer hallmarks into computer simulation models will improve their ability to predict the effects of tumor-related genes during cancer development. Indeed, some studies have used cancer hallmarks in their simulation models (Abbott *et al.*, 2006; Basanta *et al.*, 2011; Monteagudo and Santos, 2014; Spencer *et al.*, 2006). However, these hallmark simulations focused on the phenotypic (behavioral) traits of cancer cells, in which an abstract gene corresponds to a hallmark one-to-one. When a ‘phenotypic’ mutation occurs, the corresponding hallmark is affected, though in reality, one gene can contribute to multiple hallmarks and one hallmark can be affected by multiple genes. This is exemplified in the case of *TP53*, which contributes at the very least to both tumor suppression and apoptosis.

Here, we present a new computer simulation program, named *tugHall* (*tu*mor *g*ene-*Hall*mark) simulator, of a cancer-cell evolution model, wherein gene mutations are linked to the tumor cell behaviors that are influenced by the hallmarks of cancer. All first six hallmarks were implemented. Our software can utilize the accumulated genomic data to provide insights into the underlying mechanisms of cancer-cell evolution.

## 2 Model

**Fig. 1.**
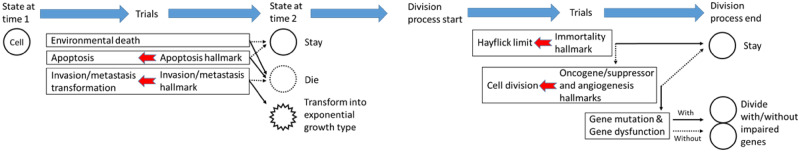
The simulation framework. A cell is put to a ‘trial’, through which the next state of the cell is probabilistically determined. For example, a cell may die in the ‘apoptosis’ trial based on the probability value for cell death by apoptosis. Hallmarks interfere with such trial probabilities when genes related to hallmarks are impaired by mutations. The variables of hallmarks represent probabilities or rates to modify trial probability values. For example, when a gene related to apoptosis is impaired, the hallmark variable of apoptosis decreases a probabilistic value in the apoptosis trial.

We briefly explain our model here, with full details provided in Supplementary Material. In the model, cells at an initial time-point are put on trials, where the next phenotypic state of each cell is probabilistically determined based on each trial. For example, a cell is put on the ‘apoptosis’ trial (Fig. 1), where the cell may die according to the ‘apoptosis’ probability variable, *a*. Cancer hallmarks are introduced to interfere with such trial probabilities. For example, the ‘evading apoptosis’, simply abbreviated to the ‘apoptosis’ hallmark (Hanahan and Weinberg, 2000), designated variable *H*_a_, decreases the apoptosis probability by *a* − *H*_a_. The value given to each cancer hallmark variable is calculated by the linear combination of gene indicator variables to represent mutational states and their constant weights (Supplementary Material). The mutational states and weights can be estimated by observed data provided by ICGC (International Cancer Genome Consortium *et al.*, 2010) and The Cancer Genome Atlas (TCGA) through ABC. Our simulation model can be interpreted as an agent-based model of branching processes where a cell’s future state is stochastically determined by trials interfered with hallmarks that are linked to gene mutations via linear combinations. The algorithm and state transitions of this model are depicted in Supplementary Material.

## 3 Results

As an example, the simulation results for colorectal cancer with the hallmarks defined in COSMIC (Forbes *et al.*, 2017) and with simple weighted coefficients has been provided. The time evolution of clones (the number of clones, number of cells in each clone, total number of cells, and final state of clones) in one simulation is shown in Supplementary Material (Supplementary Fig. S4). The simulation enabled observation of the competition between clones and determination of the clones that died. Finally, only a few clones survived. The order of gene dysfunction (from first to last) is shown with the number of cells. Additional results are described in Supplementary Material (Supplementary Results and Supplementary Fig. S5).

We have provided an illustration to show how to estimate simulation parameters by ABC and how to utilize the estimates as previously described (Kato *et al.*, 2017; Sottoriva *et al.*, 2015; Williams *et al.*, 2018). From TCGA (Ellrott *et al.*, 2018), we downloaded data on the experimentally observed VAFs of *APC*, *KRAS*, *TP53* and *PIK3CA* for a colorectal cancer patient and used the VAFs as summary statistics for ABC to estimate posterior distributions for the weight parameters (see Supplementary Material for the methods). The estimated posterior distributions suggest that *PIK3CA* makes a greater contribution to invasion/metastasis than *KRAS* and *TP53* in this patient (Supplementary Fig. S6E in Supplementary Material).

**Fig. 2.**
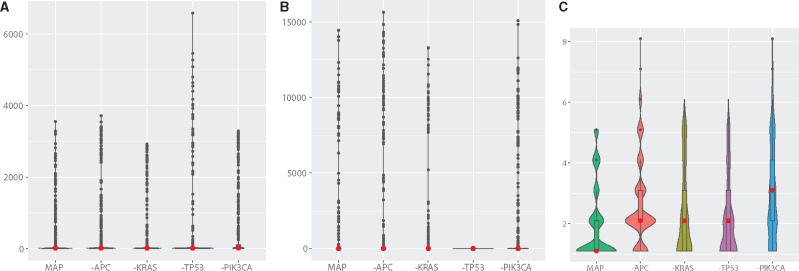
Genes influencing the numbers of primary tumor cells, metastatic cells and clones. ‘MAP’ represents simulations using MAP estimates for the weight parameters via ABC. ‘–APC’ represents simulations where the weight parameters for *APC* were set to zero and those for the other genes were kept as the MAP estimates. The same applies to the other genes. The numbers at the last time-point from 12 000 simulations are plotted as distributions. (**A**) The number of primary tumor cells. (**B**) The number of metastatic cells. (**C**) The number of clones.

Based on the maximum a posteriori probability (MAP) estimates from the posteriors, we searched for key genes important for the numbers of primary tumor cells, metastatic cells, and clones, by artificially nullifying the aberrant function of each of the four genes (i.e. by setting the weights to zero for a gene of interest). Figure 2 shows that nullifying the aberrant function of *TP53*, but not the other genes, decreased the number of metastatic cells in this patient.

## Supplementary Material

btaa182_Supplementary_Data
