# Supplementary material for: tugHall: a simulator of cancer-cell evolution based on the hallmarks of cancer and tumor-related genes
Source: Bioinformatics. 2020 Mar 14;36(11):3597–9. doi: 10.1093/bioinformatics/btaa182 (PMC7267821; doi:10.1093/bioinformatics/btaa182)

SUPPLEMENTARY DATA

tugHall: a simulator of cancer-cell evolution based on the hallmarks of cancer and tumor-related genes

Iurii Nagornov and Mamoru Kato

Department of Bioinformatics, Research Institute, National Cancer Center Japan

Supplementary Model

The value given to each cancer hallmark variable is calculated by the linear combination of gene indicator variables to represent mutational states and their constant weights (Supplementary Methods), as illustrated in Supplementary Figure 1A. For easy understanding, the simplest case is illustrated in Supplementary Figure 1B, where genes become abstracted to be hallmarks themselves by letting all hallmark variables consist of only one gene variable and by setting the weights to 1. This simplest case is equivalent to those of previous studies that focused on only phenotypic traits. The algorithm and state transitions of our model are depicted in Supplementary Figure 2.

Supplementary Methods

Hallmark variables

Trial probabilities are listed in Supplementary Table 1. Hallmark variables represent probabilities or rates to modify trial probability values as in Supplementary Table 2. The variable of hallmark x, H_x, is calculated as a linear combination of the weights, w_i^x, and the gene indicator variables, g_i^x, as follows:

$$H_x = \sum_{i=1}^{n_x} w_i^x \cdot g_i^x \tag{1}$$

$$\text{such that } \sum_{i=1}^{n_x} w_i^x = 1,\ \ 0 \le w_i^x \le 1, \tag{2}$$

$$g_i^x = \begin{cases} 1, \text{when the gene is impaired} \\ 0, \text{otherwise} \end{cases}, \tag{3}$$

where n_x represents the number of genes related to hallmark *x*. If effective trial probabilities go over 1 or under 0 due to hallmark variables, the probability values are set to 1 or 0, respectively. This modeling by linear combination is provided for simple interpretability because addition of probabilities is intuitively easy to understand.

Trials and interference by hallmarks

We here explain the trials and hallmarks listed in Supplementary Table 2

- Environmental death

A cell probabilistically dies because of various reasons, such as cell turnover (e.g., anoikis from intestinal crypts to villas), or attack by the immune system, at a constant rate, *k*.

- Apoptosis and the apoptosis hallmark

A cell probabilistically dies due to apoptosis caused by deviation from the normal state

of chromosomes, which can be caused by the accumulation of mutations. We modeled this using the sigmoid function of the proportions of impaired genes. Previous studies (Abbott, et al., 2006; Basanta, et al., 2011; Monteagudo and Santos, 2014; Spencer, et al., 2006) used a linear function, whereas a sigmoid function, with only one parameter, has a wider representation including an approximately linear function (Supplementary Figure 3). The 'evading apoptosis' (Hanahan and Weinberg, 2000), or simply 'apoptosis' hallmark decreases the probability of apoptosis through $a - H_a$, where a is the probability of apoptosis and H_a is the hallmark variable of apoptosis.

- Invasion/metastasis transformation and the invasion/metastasis hallmark

A cell probabilistically transforms into a metastatic-tumor type growth from the primary-tumor type growth form (the growth types are described later). A cell is put to this trial only when genes related to invasion/metastasis are impaired. Because it is generally thought that almost all cells die during the metastatic multi-step cascade (Weinberg, 2013), the probability of this transformation, im is set to 0 initially. This probability is increased by the hallmark of 'tissue invasion and metastasis' (Hanahan and Weinberg, 2000), or simply 'invasion/metastasis' hallmark, through $0 + H_{im}$, where H_{im} is the variable of this hallmark.

- Hayflick limit and the immortality hallmark

A cell stops cell division forever if the number of cell divisions goes over a certain threshold called the Hayflick limit. This is quantified by setting i_0, the probability of forcibly stopping cell division, to 1. However, if genes associated with Hayflick limit are impaired, a cell restores its ability to divide, which is represented by i_0 (=1) – H_i, where H_i represents the hallmark variable of 'limitless replicative potential' (Hanahan and Weinberg, 2000), or simply 'immortality'.

- Cell division, and the oncogene/suppressor and angiogenesis hallmarks

A cell probabilistically starts cell division. Cell division takes two forms: a localized growth form observed in a primary tumor, and an unlimited growth form effectively observed in a metastatic tumor. The unlimited growth form applies to cells that survive the invasion/metastasis trial; and the localized growth form applies to the other cells. We refer to the former and latter cells as the metastatic- and primary-tumor type cells, respectively.

The unlimited and localized growth forms are formalized by exponential and logistic

growth (on average across times and cells), via the effective division rate shown in Supplementary Table 2. The effective division rate for both forms consists of an initial division rate, d_0, and the incremental term + H_d. H_d is a hallmark variable that brings together two original hallmarks, "self-sufficiency in growth signal" and "insensitivity to anti-growth signals" (Hanahan and Weinberg, 2000). These two hallmarks were grouped because we concluded that both hallmarks have the same effect when genes related to these hallmarks are impaired, such as in the case of *KRAS* in the former and *RB1* in the latter. In this case, the former and latter hallmarks correspond to oncogenes and tumor suppressor genes; hence, we called this variable the 'oncogene/suppressor' hallmark variable for short.

The division rate for logistic growth is decreased by the term – $E' \times N$ (Supplementary Table 2), where E' is a variable related to carrying capacity (described below) and N is the number of surrounding (primary-tumor type) cells. The term represents a "brake" that detains the growth speed more aggressively as the number of surrounding cells increases, to simulate competition for resources. Mathematically, this logistic growth form has a carrying capacity, *i.e.*, the maximum limit for the number of cells that is determined by $1/E'$.

Carrying capacity may change when cells acquire the ability to undergo angiogenesis. This change is formalized by $E' = E_0 / (1 + F_0 * H_b)$ (Supplementary Table 2), where E_0 is an initially-given parameter and F_0 is the increase rate for H_b, which is the hallmark variable of 'sustained angiogenesis (Hanahan and Weinberg, 2000)', or simply angiogenesis. If no genes related to the angiogenesis hallmark are impaired, then the carrying capacity, $1/E'$, is $1/E_0$ because H_b is equal to 0. H_b, by definition, ranges from 0 to 1. When H_b is equal to 1, corresponding to the acquisition of full angiogenesis ability, the carrying capacity is $(1 + F_0) / E_0$, increased from 1 to $1 + F_0$ in the coefficient.

- Gene mutation

Mutations probabilistically occur following a binomial distribution along exons when a cell division occurs. Specifically, the number of mutations is randomly generated from a binomial distribution with a user-specified mutation rate in the base-pair unit and with a user-specified protein-coding gene length in the base-pair unit. Next, gene positions receiving mutations are randomly chosen from one to a specified gene length.

- Gene dysfunction

When gene positions receiving mutations are randomly determined, the mutations are probabilistically classified into those that cause gene dysfunction or not, which, in this study, are called driver or passenger mutations, respectively. Gene-dysfunction probability for oncogenes and suppressor genes can be different. This is because, for oncogenes, mutations must occur at special sites to incur gain-of-function, as typically observed in oncogenes such as *PIK3CA* (Vogelstein, et al., 2013). For suppressors, many sites can cause loss-of-function as typically observed in *RB1* (Vogelstein, et al., 2013). According to impaired genes receiving driver mutations, all hallmark variables are re-calculated to start the next iteration of simulation.

Parameter values

We will explain the possible parameter values shown in Supplementary Table 1.

- $c_{\max,0}$: Hayflick limit is considered to be 40–60, and, in culture, the Hayflick limit of fibroblasts is 50. We set it to 50 in this study.
- k_0: The probability of environmental death; it is unknown. In this study, we arbitrarily assigned it a value of 0.19, which can be changed depending on the simulation targets.
- d_0: The initial probability of division. This rate is assigned a value representing an

arbitrary time unit. We assigned 0.1, which means that one cell division occurs per ten time units on average.

- s_0: This parameter is the only parameter that changes the sigmoid function in Supplementary Table 2. The possible values in Supplementary Table 1 cover a wide range of the possible patterns of sigmoid functions, as shown in Supplementary Figure 3. Among the possible values, we assumed 10 in our simulations.
- i_0: Denotes the probability that a cell stops dividing when it uses up a finite telomere length. Here, it was assigned a value of 1.
- m_0: Parameter to define the probability of the occurrence of point mutations, which has been estimated in many studies (Preston, et al., 2010; Wang, et al., 2014; Watson, et al., 2013; Williams, et al., 2018). The possible values in Supplementary Table 1 sufficiently cover the estimated values. Here, we used 10^{-6} in the simulation of simple weighted coefficients and 10^{-5} in the simulation of approximate Bayesian computation (ABC).
- $u_{o,0}$, $u_{s,0}$: Probabilities that oncogenes and suppressor genes are impaired by point mutations, respectively. These values are unknown but the possible values in Supplementary Table 1 reasonably cover the range in orders of magnitude. For reference, the fractions of non-synonymous and nonsense changes in the codon

table are about 2/3 (66%) and 3/(64–3) (5%), respectively. We used 0.5 for $u_{o,0}$ and 0.5 for $u_{s,0}$ as the simplest case.

- E_0: This environmental variable gives the maximum number for logistic growth as $1/E_0$. The maximum distance from a capillary that a cell can survive is 0.2 mm (Weinberg, 2013), the diameter of which can contain an order of 10^3 tumor cells (Friberg and Mattson, 1997). Thus, 10^{-3} for E_0 is a reasonable estimation.
- F_0: This parameter serves to extend the maximum cell number defined by E_0 through angiogenesis. If capillaries extend 10 times in length from a main blood vessel, F_0 will become 10^3 times greater, as it is associated with volume. The possible values in Supplementary Table 1 give a sufficient range within orders of magnitude when capillaries extend by 10, 10^2, and 10^3 times their original length. Here, we used 1 for F_0 in the simulation of simple weighted coefficients and 10 in the simulation of ABC.

The other parameters:

- The number of tumor cells detected in diagnosis: 10^9 (Friberg and Mattson, 1997).
- The number of tumor cells that cause patient death: 10^{12} (Friberg and Mattson, 1997).

Simulation under simple weighted coefficients

From the COSMIC database (Forbes, et al., 2017), we extracted the relationships between hallmarks and four genes, *APC*, *KRAS*, *TP53*, and *PIK3CA*. These genes are known to cause colorectal cancer, which is a representative model of multistage tumorigenesis. According to COSMIC, *APC* is a suppressor related to H_a, H_d, H_{im}; *KRAS* is an oncogene related to H_a, H_b, H_d, H_i, H_{im}; *TP53* is a suppressor related to H_a, H_b, H_d, H_i, H_{im}; and *PIK3CA* is an oncogene related to H_a, H_b, H_d, H_{im}, where H_a, H_b, H_d, H_i, H_{im} represent the hallmarks of apoptosis, angiogenesis, oncogene/suppressor, immortality, and invasion/metastasis, respectively. For hallmark weights, we simply used equal values. Gene sizes based on the representative transcripts in COSMIC were extracted from the CCDS database (Pujar, et al., 2018). Values used for other parameters were described above in the subsection “Parameter values”.

ABC

For each hallmark weight, we sampled the values from prior distributions, assuming uniform distributions:

Each hallmark weight: {0.01, 0.1, 0.2, 0.3, 0.4, 0.5, 0.6, 0.7, 0.8, 0.9, 1.0}.

Values for the other parameters were set as described in the subsection “Parameter values.”

Under a set of parameter values, we performed a simulation to obtain the variant allele frequencies (VAFs) for the four genes, which were then used as summary statistics for ABC. We repeated the simulations 12,000 times. For experimentally-observed VAF data, we downloaded colorectal cancer data from The Cancer Genome Atlas (TCGA) database, which contained data of 508 patients. We arbitrarily selected a patient estimated to have high tumor purity based on the VAF close to 50% for *APC*, and finally obtained the VAFs of the four genes for this patient.

Using the “abc” function in “abc” package (Csillery, et al., 2012) of R, we compared the summary statistics obtained from the simulated data with those from the observed data. We used an acceptance rate of 1% to obtain the posterior distributions of the weight parameters.

Supplementary Results

The open source code is available freely in the GitHub repository

(https://github.com/nagornovys/Cancer_cell_evolution), and includes all tests and results of the testing. It also includes a program code for 100 simulation replicas and accompanying scripts to analyze the output data. The analysis script allows one to calculate the following information describing:

1) The temporal evolution of cell number for primary tumor and metastatic cells
2) The temporal evolution of the hallmarks and their probabilities
3) The number of cells vs clones defined by identical driver and all mutated genes.
4) A histogram for inequality (Gini) coefficients based on the number of cells in clones.
5) The distribution of impaired genes and the order of the genes' dysfunction.
6) The exact phylogenetic tree of clones (defined by all mutated genes) drawn by tracking the record of genetic lineages.

An example of the simulation results for colorectal cancer with the hallmarks defined in COSMIC (Forbes, et al., 2017) and with simple weighted coefficients has been provided. Supplementary Figure 4 shows the time evolution of clones (the number of clones, number of cells in each clone, total number of cells, and final state of clones) in one simulation.

Supplementary Figure 5 shows the statistical data of 100 simulation replicas: tendencies of the hallmark variables and probabilities across 100 replicas, the ID of most popular clones and distribution of number of clones at final stage, the distributions of inequality (Gini) coefficients based on the number of cells in clones defined by identical driver and all mutated genes, and the histogram of mutated genes (how many cells have mutated genes).

Supplementary Figure 6 shows the estimated posterior distributions of the weight parameters using ABC, the results of which are described in the main text.

Supplementary Figure 7 shows hallmarks influencing the number of primary tumor cells, metastatic cells, and clones. We performed 12,000 simulations under the maximum a posteriori probability (MAP) estimates from the ABC posteriors and also under only one hallmark which was artificially nullified (*i.e.*, weights for a hallmark of interest were set to zero and those for the other hallmarks were kept as the MAP estimates). We then compared the two. As a result, a lack of the apoptosis, angiogenesis, or oncogene/suppressor hallmark appeared to strongly impact the number of primary

tumor cells (Supplementary Figure 7A). A lack of the apoptosis, oncogene/suppressor, or invasion/metastasis hallmark appeared to strongly impact the number of metastatic cells (Supplementary Figure 7B).

Supplementary Discussion

Our model is extended from branching-process models (Altrock, et al., 2015) by adding the hallmark concept, and from previous hallmark models (Abbott, et al., 2006; Basanta, et al., 2011; Monteagudo and Santos, 2014; Spencer, et al., 2006) by providing a genetic perspective. Branching-process models involve only a cell division process where the cell division rate is gradually increased every time a gene is mutated. In other words, branching-process models only include the process described in the subsection "Cell division, and the oncogene/suppressor and angiogenesis hallmarks". The other hallmark phenotypes exerted by cancer-related genes, which are clearly important for understanding cancer, are not systematically included in the branching-process models.

Previous hallmark models are all-or-nothing for interference with cellular processes represented in the algorithms. For example, in the previous models (Abbott, et al., 2006; Basanta, et al., 2011; Monteagudo and Santos, 2014; Spencer, et al., 2006), cells may

die because of apoptosis but will never die from apoptosis if an apoptosis hallmark is activated. Switched-on hallmarks are interpreted as “phenotypic” (not genetic) mutations in the models. Our model connects phenotypic changes through genetic mutations. For example, cells gradually increase the probability of resistance against apoptosis each time one of the apoptosis-related genes is mutated. Thus, our model can connect the phenotypic nature of cancer cells with experimentally observed gene mutation data, as demonstrated in the ABC application. Previous hallmark models did not utilize experimentally observed data. Furthermore, as illustrated in Supplementary Figure 1, our model includes a previous hallmark model as a special case when every hallmark variable in our model is related to only one gene and all weights are set to one (under the one phenotype-one gene assumption).

Supplementary Declarations

Ethics approval and consent to participate

Not applicable

Authors' contributions

MK designed the study, developed the model, and wrote the manuscript. NI designed the program code and tests and made simulations.

References

Abbott, R.G., Forrest, S. and Pienta, K.J. Simulating the hallmarks of cancer. *Artif Life* 2006;12(4):617-634.

Altrock, P.M., Liu, L.L. and Michor, F. The mathematics of cancer: integrating quantitative models. *Nat Rev Cancer* 2015;15(12):730-745.

Basanta, D., *et al.* Computational analysis of the influence of the microenvironment on carcinogenesis. *Math Biosci* 2011;229(1):22-29.

Csillery, K., Francois, O. and Blum, M.G.B. abc: an R package for approximate Bayesian computation (ABC). *Methods in Ecology and Evolution* 2012;3(3):475-479.

Forbes, S.A., *et al.* COSMIC: somatic cancer genetics at high-resolution. *Nucleic Acids Res* 2017;45(D1):D777-D783.

Friberg, S. and Mattson, S. On the growth rates of human malignant tumors: implications for medical decision making. *J Surg Oncol* 1997;65(4):284-297.

Hanahan, D. and Weinberg, R.A. The hallmarks of cancer. *Cell* 2000;100(1):57-70.

Monteagudo, A. and Santos, J. Studying the capability of different cancer hallmarks to initiate tumor growth using a cellular automaton simulation. Application in a cancer stem cell context. *Biosystems* 2014;115:46-58.

Preston, B.D., Albertson, T.M. and Herr, A.J. DNA replication fidelity and cancer. *Semin*

Cancer Biol 2010;20(5):281-293.

Pujar, S., *et al.* Consensus coding sequence (CCDS) database: a standardized set of human and mouse protein-coding regions supported by expert curation. *Nucleic Acids Res* 2018;46(D1):D221-D228.

Spencer, S.L., *et al.* Modeling somatic evolution in tumorigenesis. *PLoS Comput Biol* 2006;2(8):e108.

Vogelstein, B., *et al.* Cancer genome landscapes. *Science* 2013;339(6127):1546-1558.

Wang, Y., *et al.* Clonal evolution in breast cancer revealed by single nucleus genome sequencing. *Nature* 2014;512(7513):155-160.

Watson, I.R., *et al.* Emerging patterns of somatic mutations in cancer. *Nat Rev Genet* 2013;14(10):703-718.

Weinberg, R. The biology of cancer. Garland science; 2013.

Williams, M.J., *et al.* Quantification of subclonal selection in cancer from bulk sequencing data. *Nat Genet* 2018;50(6):895-903.

Supplementary Figure Legends

Supplementary Figure 1. Hallmark variables.

(A) Illustration of hallmark variables by the oncogene/suppressor hallmark variable, H_d. A hallmark variable is defined as a linear combination of weights and gene indicator variables, where weights are real numbers ranging from 0 to 1 such that the summation is equal to 1. Gene indicator variables have a value of 1 if a gene is impaired; otherwise, they have a value of 0. In the illustration, four genes are associated with the oncogene/suppressor hallmark through such arbitrary weights, and the first two genes are impaired, which influences the hallmark. The total influence is calculated as H_d = 0.3. Eventually, the probability of the cell division trial, *d'*, is increased by this amount, 0.3, to enhance cell division. (B) The simplest model. The number of weight for each hallmark is one and the weight value is one. The indicator variable, *I*, represents whether a hallmark itself is impaired (I =1) or otherwise (I =0).

Supplementary Figure 2. Synopsis of our model.

(A) Pseudocode of our algorithm. (B) The state transitions of the model: lines are transitions between trials with related probabilities (*k*, *a*', *im*', etc.; see Supplementary

Table 1 and Supplementary Table 2), and the light blue area contains the processes and states related to cell division. The START state is represented by the circle in green.

Supplementary Figure 3. Shapes of a sigmoid function with only one parameter being changed.

The x-axis represents the proportion of impaired genes, and s_0 is the parameter.

Supplementary Figure 4. The results of a single simulation.

Time evolution of: (A) the number of primary tumor and metastatic cells; (B) the average probabilities d', i', im', a, k; (C) the average hallmark variables $H_d, H_i, H_{im}, H_a, H_b$; (D) the number of cells in each clone with the inset of this plot for small clones; (E) the number of clones; and (H) inequality (Gini) coefficients based on the number of cells in each clone defined by identical driver genes or by identical all mutated genes. (F and G) The number of cells at the last time step for each clone defined by identical driver genes or identical all-mutated genes. (I) The order of gene dysfunction (from first to last) for each clone. (J) Phylogenetic tree drawn by tracking back the records of parental cells for cell clones (cells can be compressed into a clone because individual cells in a clone have an identical mutation code). The tips represent

clone IDs and circles in the nodes represent the clone from which successive clones were born. The lines (with colors) represent the time step when the clones were born.

Supplementary Figure 5. The results for 100 simulation replicas.

Time evolution for (A) the number of primary tumor and metastatic cells, (B) the average probabilities d', i', im', a, k, and (C) the average hallmark variables $H_d, H_i, H_{im}, H_a, H_b$. The distributions across 100 replicas for (D and E) number of clones and (F) inequality (Gini) coefficients (for drivers and for drivers plus passengers). (G and H) The number of cells in the most popular clones. (I) The fraction of mutated genes, (J) the number of cells with mutated genes for each gene, and (K) the number of cells with 1, 2, 3, or 4 mutated genes.

Supplementary Figure 6. Estimated posterior distributions of the weight parameters of tumor-related genes in the ABC analysis.

The lines in red represent the MAP estimates. (A) Apoptosis hallmark. (B) Angiogenesis hallmark. (C) Oncogene/suppressor hallmark. (D) Immortality hallmark. (E) Invasion/metastasis hallmark.

Supplementary Figure 7. Hallmarks influencing the numbers of primary tumor cells, metastatic cells, and clones.

“MAP” represents simulations using MAP estimates for the weight parameters in the ABC analysis. “–Ha” represents simulations in which the weight parameters for the apoptosis hallmark were set to zero and those for the other hallmarks were kept as the MAP estimates. The same applies to the other hallmarks, where Hb, Hd, Hi, and Him represent the hallmarks of angiogenesis, oncogene/suppressor, immortality, and invasion/metastasis, respectively. The numbers at the last time-point from 12,000 simulations are plotted as distributions. (A) Number of primary tumor cells. (B) Number of metastatic cells. (C) Number of clones.

Supplementary Tables

Supplementary Table 1. The variables.

Variable type	Notation	Description	Per	Interfered by hallmarks	Time change	Notation as parameter	Possible initial values for parameter
Cell	c	Cell division counter	-	No	Dynamic	-	-
	c_{max}	Maximum cell division number by Hayflic limit	-	No	Static	($c_{max,0}$)	50
	k	Probability of cell death by environments	τ	No	Static	k_0	{0.1, 0.2, ..., 0.9}
	d	Cell division rate	τ	Yes	Dynamic	(d_0)	0.1 for τ, arbitrarily time unit
	im	Probability of invasion/metastasis transformation	τ	Yes	Dynamic	-	-
	a	Probability of cell death by apoptosis	τ	Yes	Dynamic	s_0	{10, 15, 20, 30, 40, 90}
	i	Probability of cell division stop by Hayflic limit	τ	Yes	Dynamic	(i_0)	1
	m	Mutation rate per bp	division	No	Static	m_0	{**10^{-6}**, 10^{-7}, 10^{-8}, **10^{-9}**, **10^{-10}**}
	u_o	Probability of dysfunction of a oncogene	mutation	No	Static	$u_{o,0}$	{1/1, 1/10, 1/100}
	u_s	Probability of dysfunction of a suppressor	mutation	No	Static	$u_{s,0}$	{1/1, 1/10, 1/100}
External	N/M	Number of cells with logistic/exponential growth	-	No	Dynamic	-	-
	E	Environmental resource limitation	-	No	Static	E_0	{10^{-1}, 10^{-2}, **10^{-3}**, 10^{-4}, 10^{-5}}
	F	Reduction effect to E by angiogenesis	-	Yes	Static	F_0	{10^1, 10^2, 10^3, 10^6}
	T	Time counter	-	-	-	-	-

Cell variables in the "variable type" column are those attributed to each single cell and inherited by a daughter cell in cell division.

External variables are those that represent the outside environments of cells. The “Per” column represents units by which the rates or probabilities are defined. τ is an arbitrary time unit; for example, d_0 can be set to 0.1 such that a cell division occurs per 10τ on average. Dynamic and static variables in the “time change” column denote whether values change or not with time, respectively. The “notation as parameter” column indicates notations where initial values have to be specified to start a simulation. The parentheses represent initial values that are reasonably estimated or specified as one value. Together, this means that the number of free parameters in this system is only 7. s_0 for *a* denotes the gain parameter for a sigmoid function, the specific formula for which is given in Supplementary Table 2. In the “possible initial values” column, the underlined values in bold are supported by the literature for m_0 (Preston, et al., 2010; Wang, et al., 2014; Watson, et al., 2013; Williams, et al., 2018) and E_0 (Friberg and Mattson, 1997; Weinberg, 2013).

Supplementary Table 2. The trials.

Trials	Condition	Probability	Event
Environmental death	Every time step	• $k' = k_0$	Death
		• $1 - k'$	Nothing
Apoptosis	Every time step	• $a' = a - H_a = \sigma(s_0 \times (x - 0.5)) - H_a$, where $x = impaired\_gene\_density$	Death
		• $1 - a'$	Nothing
Invasion/ metastasis transformation	$im' \neq 0$	• $im' = H_{im} < 1$	Nothing
		• $im' = H_{im} = 1$	Transform into exponential growth
		• $1 - im'$	Death
Hayflick limit (immortalization)	$c > c_{max}$	• $i' = i_0 - H_i$	Stop division process
		• $1 - i'$	Start division trial
Cell division	Every time step	• $d' = \begin{cases} d - E' \times N, \text{ when logistic growth} \\ d, \text{ when exponential growth} \end{cases}$, where $d = d_0 + H_d$ and $E' = E_0 / (1 + F_0 \times H_b)$	Division
		• $1 - d'$	Nothing
Gene mutation	Cell division happens	• $m' = m_0 \times CDS\_length$	Mutation
		• $1 - m'$	Nothing
Gene dysfunction	Gene mutation happens	• $u' = \begin{cases} u_{o,0}, \text{ for oncogene} \\ u_{s,0}, \text{ for suppressor} \end{cases}$	Gene dysfunction
		• $1 - u'$	Nothing

The "condition" column represents the condition in which the trial is applied. Variables

with prime (‘) in the “probability” column are effective (actually used) probabilities. *H*s represent hallmark variables. σ is a sigmoid function. *Impaired_gene_density* represents the fraction of impaired genes.

Supplementary Figure 1

A The oncogene/suppressor hallmark variable, H_d, for example:

$$H_{\mathrm{d}} = w_1^{\mathrm{d}} \cdot g_1^{\mathrm{d}} + w_2^{\mathrm{d}} \cdot g_2^{\mathrm{d}} + w_3^{\mathrm{d}} \cdot g_3^{\mathrm{d}} + w_4^{\mathrm{d}} \cdot g_4^{\mathrm{d}}$$

Let $(w_1^{\mathrm{d}}, w_2^{\mathrm{d}}, w_3^{\mathrm{d}}, w_4^{\mathrm{d}}) = (0.1,\ 0.2,\ 0.3,\ 0.4)$

When $(g_1^{\mathrm{d}}, g_2^{\mathrm{d}}, g_3^{\mathrm{d}}, g_4^{\mathrm{d}}) = (1,\ 1,\ 0,\ 0)$

$$H_{\mathrm{d}} = 0.1 \cdot 1 + 0.2 \cdot 1 + 0.3 \cdot 0 + 0.4 \cdot 0 = 0.3$$

H_d interferes with a probability value of the cell division trial:

$$d' = d_0 + H_{\mathrm{d}} = 0 + 0.3 = 0.3$$

B The simplest model

$$\begin{bmatrix} H_{\mathrm{a}} \\ H_{\mathrm{im}} \\ H_{\mathrm{i}} \\ H_{\mathrm{d}} \\ H_{\mathrm{b}} \end{bmatrix} = \begin{bmatrix} w_1^{\mathrm{a}} \cdot g_1^{\mathrm{a}} \\ w_1^{\mathrm{im}} \cdot g_1^{\mathrm{im}} \\ w_1^{\mathrm{i}} \cdot g_1^{\mathrm{i}} \\ w_1^{\mathrm{d}} \cdot g_1^{\mathrm{d}} \\ w_1^{\mathrm{b}} \cdot g_1^{\mathrm{b}} \end{bmatrix} = \begin{bmatrix} 1 \cdot I_{\mathrm{a}} \\ 1 \cdot I_{\mathrm{im}} \\ 1 \cdot I_{\mathrm{i}} \\ 1 \cdot I_{\mathrm{d}} \\ 1 \cdot I_{\mathrm{b}} \end{bmatrix}$$

Supplementary Figure 2

A

Algorithm 1: tugHall algorithm

Initialization: $i_{step} = 1$; Objects: **cells, genes, hallmarks**, including:
Input: read the names of onco genes from ”gene_cds2.txt” - initialization of **genes**
Input: read the weights for hallmarks from ”gene_cds2.txt” - initialization of **hallmarks**
Input: read the initial cells from ”cellinit.txt” - initialization of **cells**
Output: Save input data into the files: log.txt, Weights.txt and geneout.txt
while $(0 < N_{cells} < N_{cells\_max})$ and $(i_{step} < N_{steps\_max})$ **do**
  trial each cell for several processes using $trial()$ function
  begin
    • **environmental** death with probability k
    • **apoptosis** death with probability a'
    • **invasion/metastasis** transformation with probability im'
    • **Hayflick limit** with probability i'
    • **division** process with probability d'
    return 0 - death, 1 - save, 2 - division
  end
  duplicate the cells to divide
  mutagenesis trial of the parents and daughters cells independently using $trial\_mutagenesis()$ function
  begin
    • gene mutation with probability m'
    • **if** (mutation is happened) **then**
      choose the gene/genes and position on it
      gene dysfunction with probability u': passenger or driver mutation
    end
  end
  delete cells which died
  calculate new values of the hallmarks and probabilities for all cells
  update Environment variables: calculate the average values of the probabilities and hallmarks, N_{cells}
  Output: write cells, environment, hallmarks and genes data to ”cellout.txt” file for i_{step} time step
  next step: $i_{step} = i_{step} + 1$
end

Supplementary Figure 2 (contd.)

B

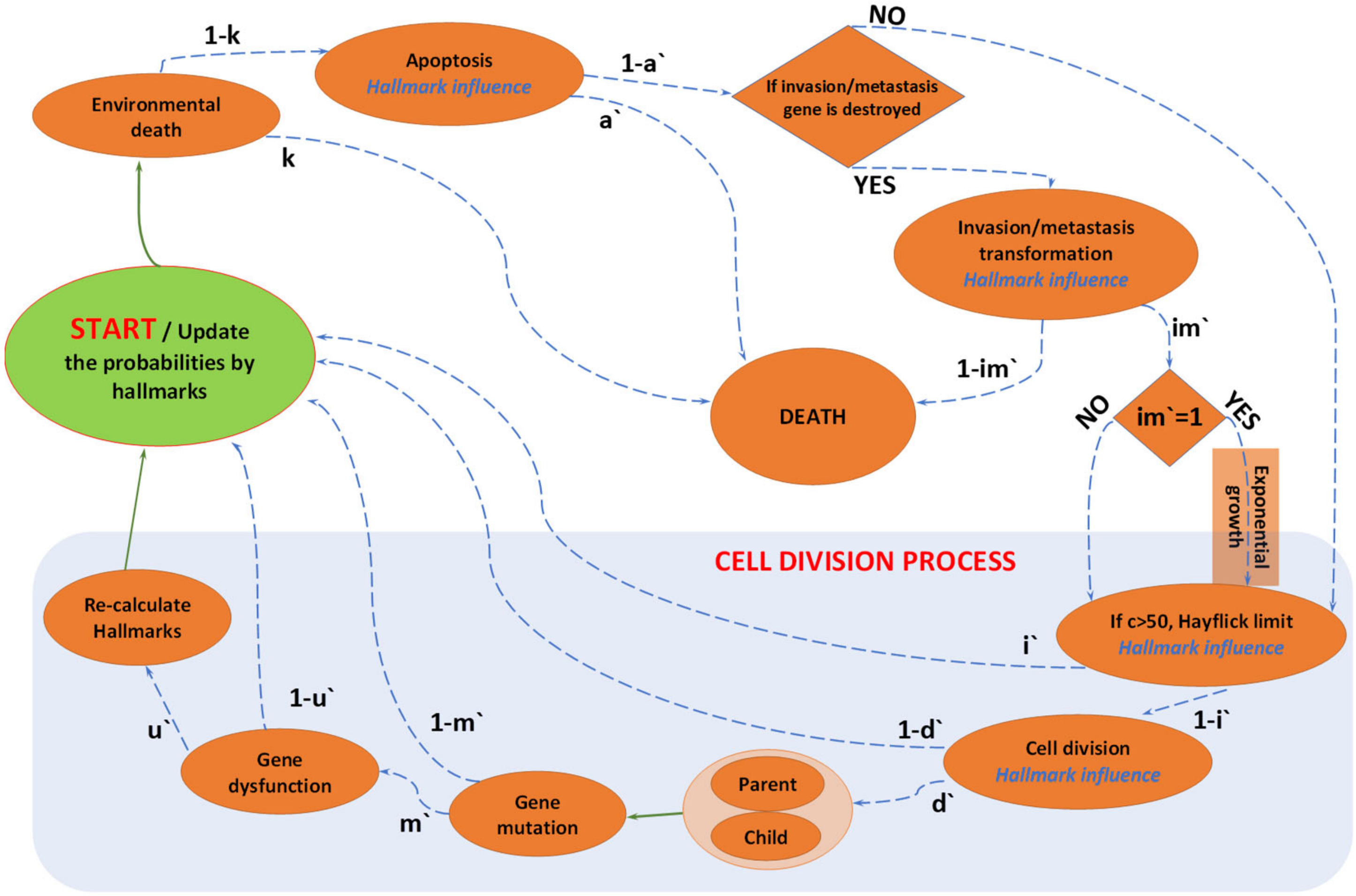

Supplementary Figure 3

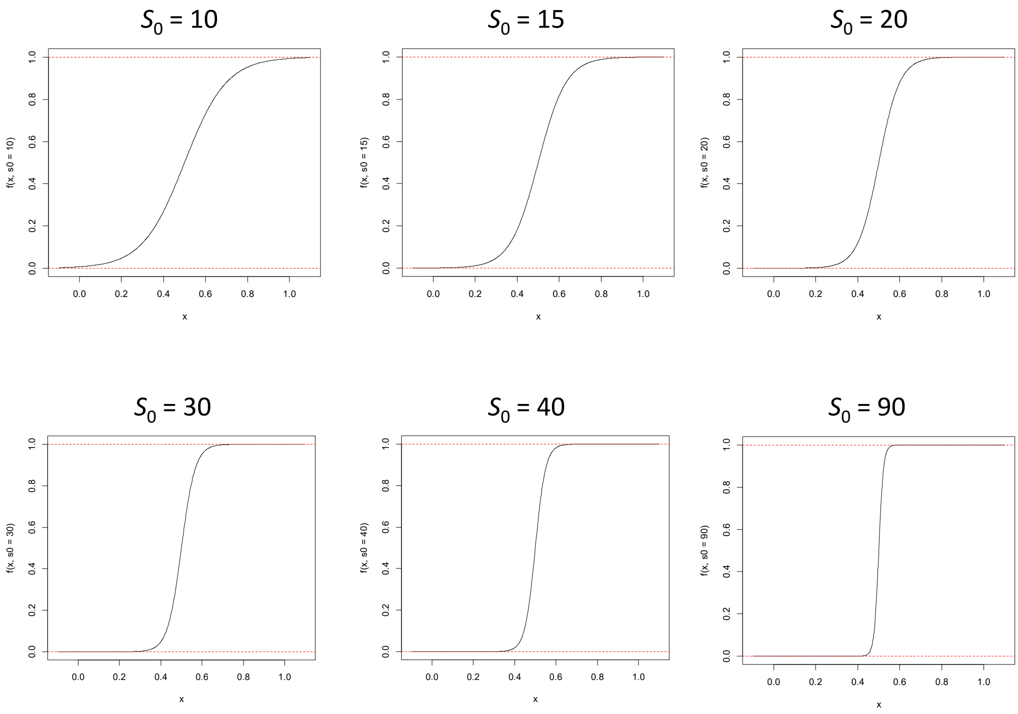

Supplementary Figure 4

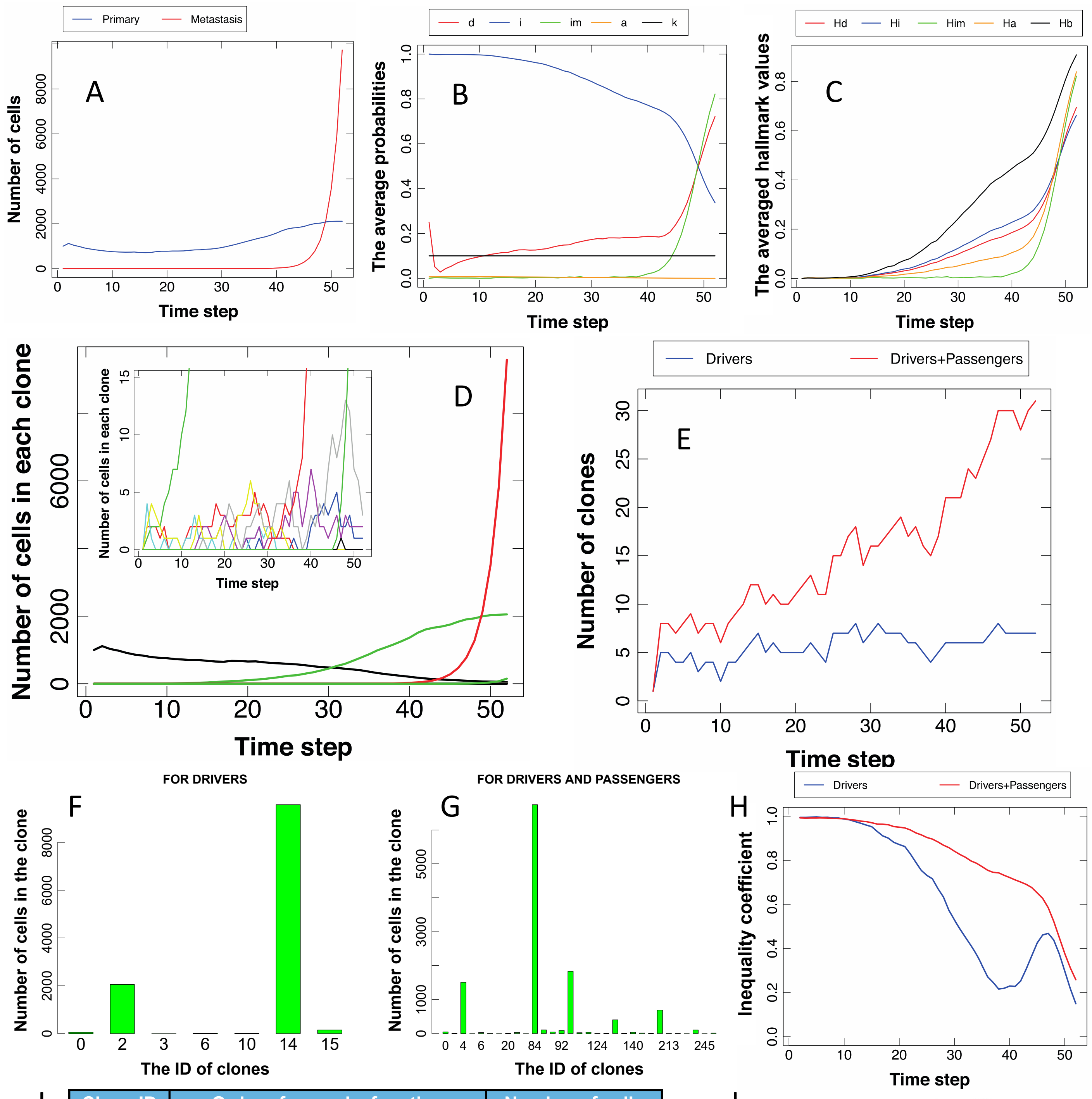

I

Clone ID	Order of gene dysfunction	Number of cells
14	TP53 -> APC -> KRAS	9581
2	TP53	2050
15	TP53 -> APC -> KRAS -> PIK3CA	151
0	No dysfunction	54
10	TP53 -> APC	3
6	TP53 -> KRAS	2
3	TP53 -> PIK3CA	1

J

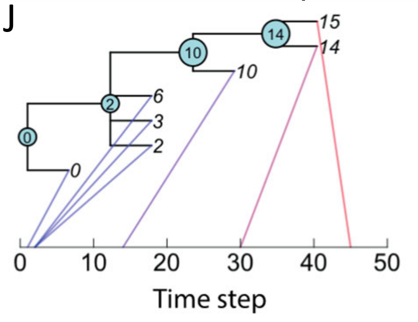

Supplementary Figure 5

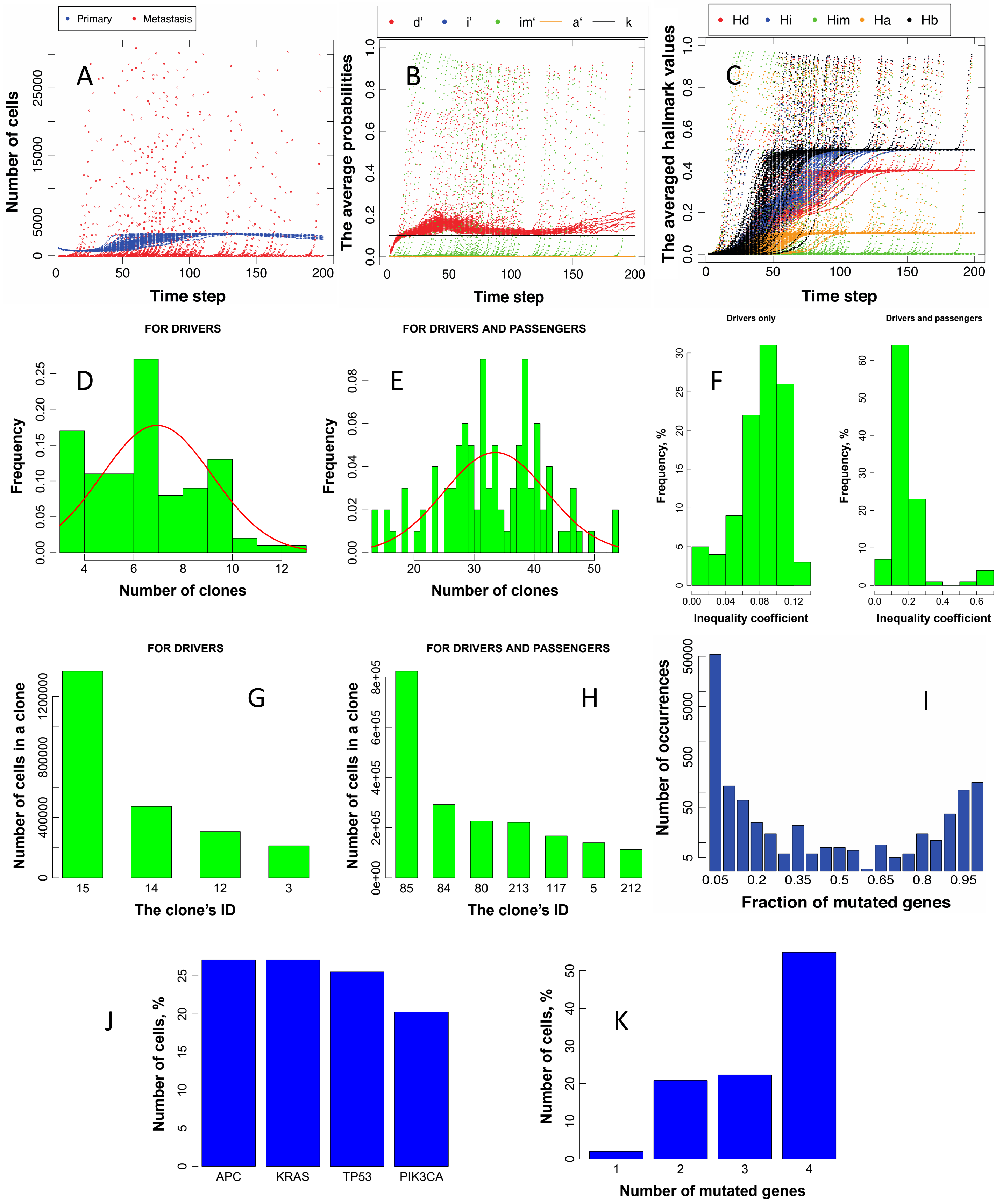

Supplementary Figure 6

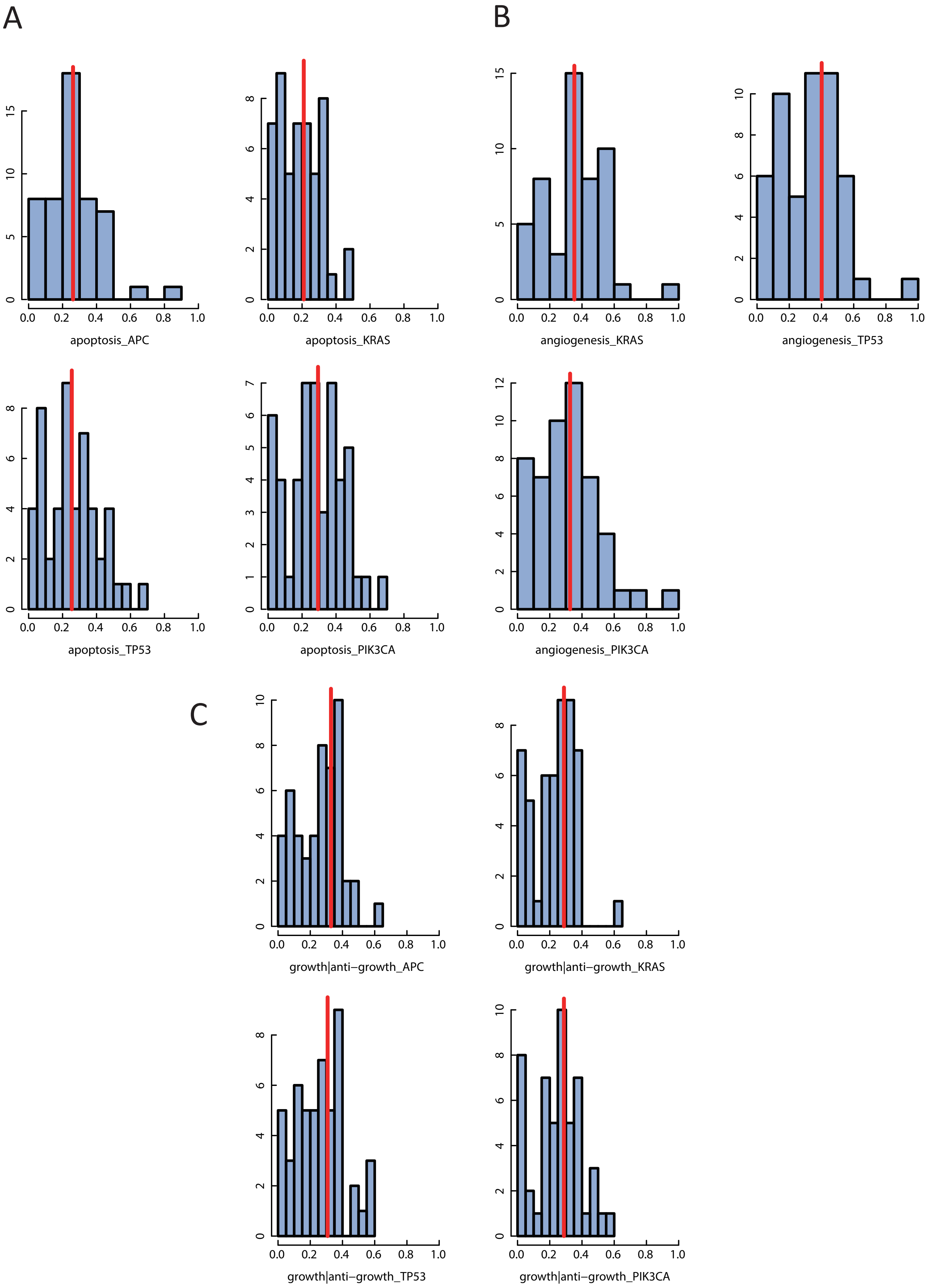

Supplementary Figure 6 (contd.)

D

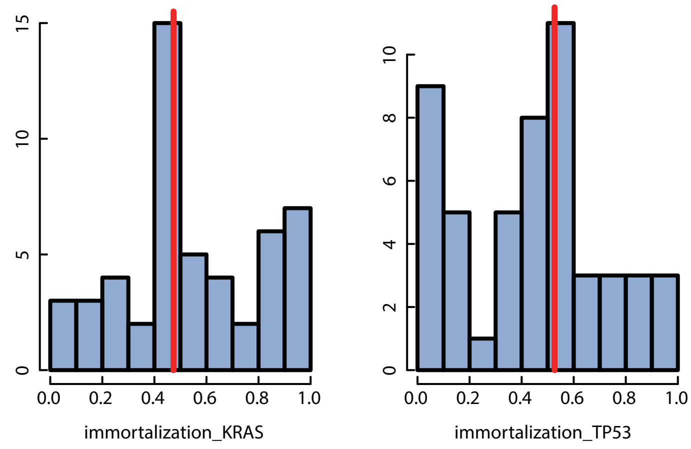

E

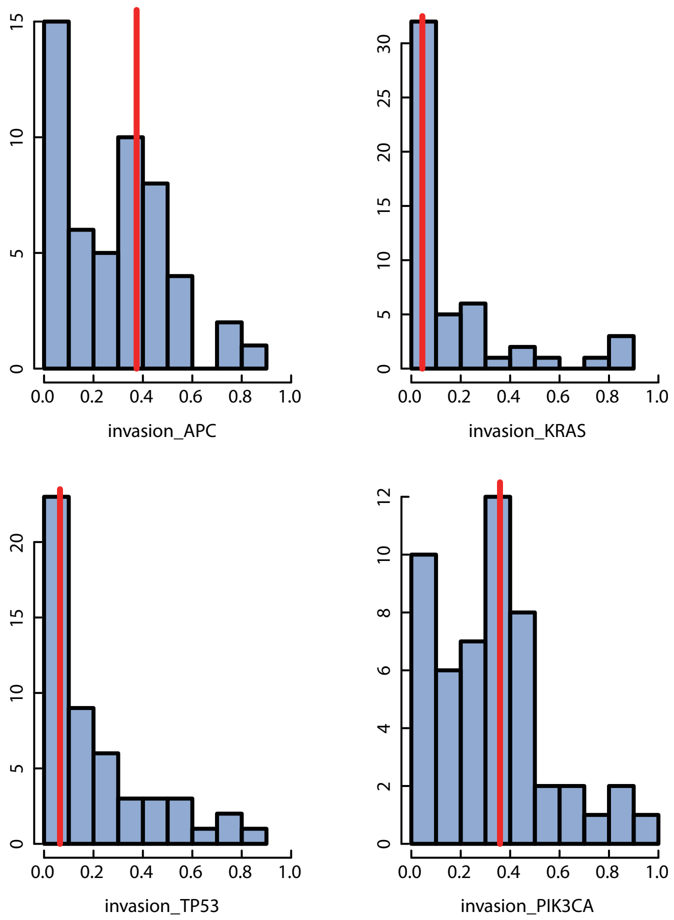

Supplementary Figure 7

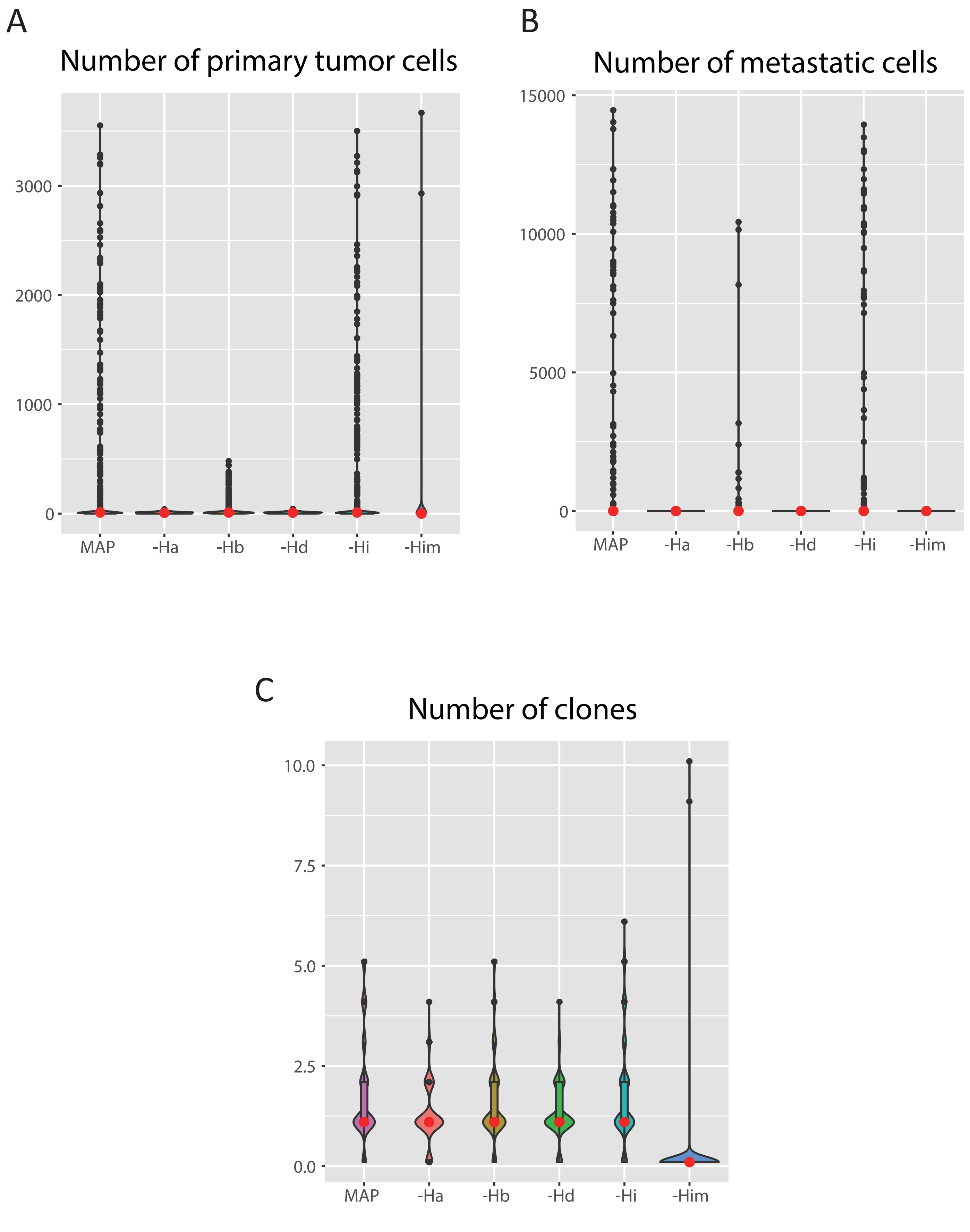

Supplement: btaa182_Supplementary_Data [file btaa182_supplementary_data.pdf]
